# Prevenção Farmacológica Secundária da Doença Arterial Coronariana em Pacientes Submetidos ao Manejo Clínico, Intervenção Coronária Percutânea ou Cirurgia de Revascularização Miocárdica

**DOI:** 10.36660/abc.20220403

**Published:** 2023-02-16

**Authors:** Marcelo B. Lucca, Felipe C. Fuchs, Adriana S. Almeida, Marco V. Wainstein, Flavio D. Fuchs, Sandra C. Fuchs

**Affiliations:** 1 Programa de Pós-graduação em Cardiologia Faculdade de Medicina Universidade Federal do Rio Grande do Sul Porto Alegre RS Brasil Programa de Pós-graduação em Cardiologia , Faculdade de Medicina , Universidade Federal do Rio Grande do Sul , Porto Alegre , RS – Brasil; 2 Instituto Nacional de Ciência e Tecnologia PREVER Centro de Pesquisa Clínica Hospital de Clínicas de Porto Alegre Porto Alegre RS Brasil Instituto Nacional de Ciência e Tecnologia PREVER – Centro de Pesquisa Clínica - Hospital de Clínicas de Porto Alegre , Porto Alegre , RS – Brasil; 3 Divisão de Cardiologia Hospital de Clínicas de Porto Alegre Universidade Federal do Rio Grande do Sul Porto Alegre RS Brasil Divisão de Cardiologia – Hospital de Clínicas de Porto Alegre , Universidade Federal do Rio Grande do Sul , Porto Alegre , RS – Brasil

**Keywords:** Doença Arterial Coronariana, Ponte de Artéria Coronária, Intervenção Coronária Percutânea, Prevenção secundária, Tratamento farmacológico

## Abstract

**Fundamento:**

A prevenção secundária é recomendada a pacientes com evidência de doença arterial coronariana (DAC) independentemente da indicação de tratamento por cirurgia de *bypass* da artéria coronária (CABG) ou intervenção coronária percutânea (ICP).

**Objetivos:**

Este estudo avaliou se o tratamento clínico, a ICP ou o CABG teve influência na adesão à prevenção secundária farmacológica em pacientes com DAC estável.

**Métodos:**

Esta coorte incluiu pacientes com idade ≥40 anos com DAC estável confirmada por angiografia coronária estável. A decisão por tratamento clínico isolado, ou combinado com ICP ou CABG foi feita por médicos assistentes. A adesão às drogas prescritas recomendadas pelas diretrizes de prevenção secundária (tratamento farmacológico ótimo), incluindo agentes antiplaquetários, drogas hipolipemianetes, betabloqueadores, e bloqueadores do sistema angiotensina aldosterona, foi avaliada no acompanhamento. Diferenças com valores de p < 0,05 foram consideradas estatisticamente significativas.

**Resultados:**

Dos 928 pacientes incluídos inicialmente, 415 apresentaram DAC leve e 66 apresentaram DAC leve a moderada. O período médio de seguimento foi 5,2 ± 1,5 anos. Os pacientes submetidos ao CABG apresentaram maior probabilidade de receberem tratamento farmacológico ótimo que aqueles submetidos à ICP ou tratamento clínico (63,5% versus 39,1% versus 45,7% respectivamente, p=0,003). Fatores basais independentemente associados com maior probabilidade de prescrição de tratamento ótimo foram CABG [39% maior (6% - 83%, p=0,017)] em comparação a outros tratamentos e diabetes [25% maior (1% - 56%), p=0,042] em comparação à ausência de diabetes.

**Conclusões:**

Pacientes com DAC submetidos ao CABG são mais frequentemente tratados com prevenção secundária farmacológica ótima que pacientes tratados com ICP ou exclusivamente com tratamento clínico.

**Figura Central:**
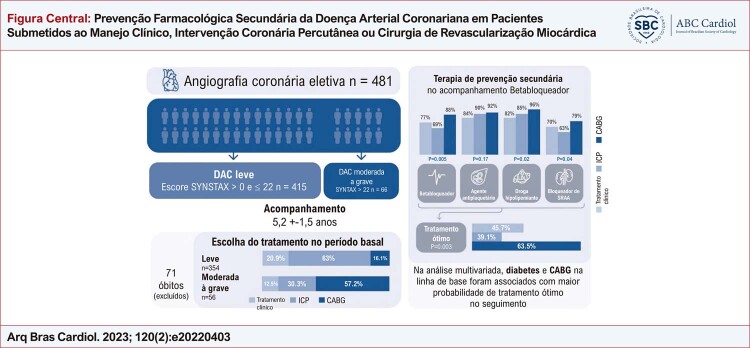
: Prevenção Farmacológica Secundária da Doença Arterial Coronariana em Pacientes Submetidos ao Manejo Clínico, Intervenção Coronária Percutânea ou Cirurgia de Revascularização Miocárdica

*DAC: doença arterial coronariana; ICP: intervenção coronária percutânea; CABG: bypass da artéria coronária.*

## Introdução

A doença cardiovascular tem sido a principal causa de morte e peso de doença no mundo nos últimos 15 anos. ^1^ A doença arterial coronariana (DAC), uma de suas apresentações, afeta entre 5% e 8% dos brasileiros com idade superior a 40 anos. Pacientes com manifestações clínicas de DAC, tais como angina pectoris, infarto do miocárdio, e evidência de lesões na angiografia coronária são candidatos à prevenção secundária. ^2^ As diretrizes recomendam o uso de agentes antiplaquetários, drogas hipolipemiantes, betabloqueadores e bloqueadores do sistema renina-angiotensina-aldosterona (SRAA), todos com altos níveis de evidência. ^3 - 5^

A revascularização realizada por *bypass* da artéria coronária (CABG) melhora a sobrevida de pacientes com doença da artéria coronária principal esquerda desprotegida, DAC de três vasos, ou diabetes, principalmente para aqueles com sintomas graves, testes não invasivos positivos precoces, ou disfunção do ventrículo esquerdo. A intervenção coronária percutânea (ICP) é geralmente a opção de escolha para indivíduos sem uma indicação clara para CABG, cujos sintomas persistem apesar do tratamento farmacológico. ^3^ Ensaios randomizados grandes comparando estratégias conservadoras invasivas iniciais para pacientes com DAC estável não encontraram diferenças significativas em eventos cardiovasculares ou mortalidade. ^6 - 9^

Independentemente de revascularização, o manejo farmacológico continua sendo o tratamento padrão para prevenção secundária da DAC. ^3 - 5^ Contudo, a adesão tem sido subótima em muitos contextos. ^10 - 12^ No Euro Heart Study, uma proporção considerável de indivíduos com DAC estável em tratamento clínico ou invasivo não estava em tratamento farmacológico ótimo, o que foi associado a piores desfechos. ^13^ Uma análise pós-hoc do estudo “ *Synergy Between PCI with Taxus and Cardiac Surgery (SYNTAX)* ” mostrou ainda que a proporção de pacientes que receberam tratamento farmacológico ótimo foi de 41% na alta de revascularização, e caiu para um terço após cinco anos. ^14^ Em uma metanálise de estudos sobre revascularização coronariana, o tratamento farmacológico ótimo reduziu de 40% em um ano de seguimento para 38% em cinco anos, e as porcentagens de ICP permaneceram mais altas que as de CABG em todos os momentos. ^15^ Os dados também sugerem uma correlação entre as diferenças na adesão e desfechos clínicos ao se comparar ICP com CABG em cinco anos. Um estudo conduzido no Brasil detectou diferenças no tratamento farmacológico entre indivíduos de baixa renda e indivíduos de alta renda. ^16^ Em nosso conhecimento, a associação entre o tipo de tratamento, CABG, e ICP, ou exclusivamente a adesão à prevenção secundária não foram avaliadas em uma coorte contemporânea. O objetivo deste estudo foi avaliar se o método de tratamento de DAC estável – CABG, ICP, ou tratamento clínico exclusivo – influenciou a adesão ao tratamento farmacológico ótimo para prevenção secundária da DAC.

## Métodos

Os participantes deste artigo foram avaliados em um estudo do tipo coorte delineado para avaliar vários desfechos em pacientes com DAC estável. ^17 , 18^ A distribuição de mortes e outros eventos cardiovasculares maiores (MACE) no acompanhamento, segundo o tratamento inicial, foi descrito anteriormente. ^17 , 18^

A coorte incluiu homens e mulheres com idade ≥ 40 anos, com DAC estável e significativa na angiografia. Os pacientes foram encaminhados para angiografia coronária eletiva devido à suspeita clínica de DAC ^19^ com ou sem evidência de isquemia em testes não invasivos. No basal, indivíduos com síndrome coronariana aguda, revascularização prévia (CABG ou ICP), doença renal crônica, diagnóstico atual ou prévio de câncer, doença psiquiátrica grave, ou nenhuma evidência de DAC significativa [escore SYNTAX (SXscore) <1] foram excluídos.

Fatores de risco cardiovasculares tradicionais, fatores socioeconômicos e demográficos, estilo de vida, e dados prévios de morbidade foram avaliados no período basal durante uma entrevista presencial usando um questionário padrão. Assistentes treinados realizaram as medidas de pressão arterial e medidas antropométricas na inclusão, antes da realização do cateterismo. A hipertensão foi definida como pressão arterial sistólica (PAS) ≥ 140 mmHg, pressão arterial diastólica (PAD) ≥ 90 mmHg, ou uso de agentes anti-hipertensivos. O Índice de Massa Corporal (IMC) [peso (Kg)/altura (m ^2^ )] foi categorizado como < 25, 25-29, ou ≥30 kg/m ^2^ .

As avaliações laboratoriais foram avaliadas 12 horas após jejum. As amostras de sangue foram colhidas da bainha femoral imediatamente após a inserção do cateter cardíaco e antes da administração de heparina. Diabetes mellitus foi definido como glicemia de jejum ≥126 mg/dL ou uso de agentes hipoglicemiantes. Hipercolesterolemia foi caracterizada por níveis de colesterol total ≥200 mg/dL ou uso de drogas hipolipemiantes. A angiografia coronária no cateterismo foi realizada por cardiologistas intervencionistas experientes por acessos radial e transfemoral. DAC significativa foi diagnosticada por análise quantitativa de vasos epicárdicos principais (por exemplo artéria coronária principal esquerda, artéria descendente anterior, artéria circunflexa, artéria coronária direita, e vasos com diâmetros ≥2,5 mm), ramos diagonais, artéria marginal obtusa, ramo posterolateral, e artéria descendente posterior. ^17^ DAC significativa foi definida pela presença de pelo menos uma artéria coronária epicárdica principal com estenose ≥50%. O escore SXscore foi calculado para cada artéria afetada, e os escores adicionados para se obter o SXscore final do paciente. ^20^ Um SXscore ≤ foi categorizado como DAC leve e escores maiores que 22 foram categorizados como DAC moderada à grave. ^21^ Dois cardiologistas intervencionistas avaliaram independentemente uma subamostra de imagens, e o controle de qualidade foi feito por um terceiro médico que avaliou a variação entre observadores. Os médicos assistentes que não participaram do estudo receberam as imagens e um laudo da angiografia coronária, mas desconheciam os valores do SXscores. A decisão entre CABG, ICP, ou tratamento clínico exclusivo foi definida pelos médicos assistentes, com base em treinamento prévio em cardiologia, mas prática clínica usual não padronizada. Os médicos assistentes geralmente discutiam casos complexos com cardiologistas e cirurgiões intervencionistas que conduziram o procedimento diagnóstico.

No seguimento, os pacientes foram convidados por telefone para serem entrevistados por um médico treinado. Utilizou-se um questionário padronizado para registrar o tratamento realizado após a angiografia coronária inicial, comorbidades subsequentes, admissões hospitalares, tratamento médico atual, e estado geral de saúde. A adesão ao tratamento farmacológico ótimo foi definida como o uso relatado pelo paciente de todas as medicações recomendadas para a prevenção secundária a que os pacientes eram elegíveis, incluindo agentes antiplaquetários, estatinas ou outras drogas hipolipemiantes, betabloqueadores, e bloqueadores do SRAA, fornecidas pelo sistema público de saúde.

### Tamanho amostral e análise estatística

O tamanho amostral foi calculado para testar a hipótese primária, ^17^ com poder de 80% e nível de significância de 0,05 (bicaudal) para detectar uma razão de chance ( *hazard ratio* ) de pelo menos 2,4, considerando que 5% dos pacientes com um SXscore baixo e 12% com um SXscore alto apresentaria MACE. Nessa análise adicional, incluímos pacientes sobreviventes que apresentaram um SXscore > 0 e foram, portanto, elegíveis para prevenção secundária. Todas as análises foram conduzidas pelo programa *Statistical Package for the Social Sciences* (SPSS; versão 22.0; IBM corp., Armonk, NY, EUA). Diferenças foram consideradas significativas se apresentassem um valor de p <0,05. A normalidade dos dados foi verificada usando o *boxplot* e o teste de Shapiro-Wilk. As variáveis contínuas foram apresentadas em média ± desvio padrão (DP), e as variáveis categóricas em números absolutos, com porcentagens e intervalos de confiança quando relevante. As características basais foram analisadas usando a análise de variância (one-way ANOVA com correção de Bonferroni) para variáveis contínuas e o teste do qui-quadrado para variáveis categóricas para comparar tratamento clínico, ICP e CABG. O teste do qui-quadrado foi usado para avaliar a proporção de pacientes tratados com tratamento farmacológico ótimo entre pacientes tratados com terapia clínica exclusiva com aqueles tratados também com CABG ou ICP. Ainda, avaliamos a associação de várias características basais com a adesão à prevenção secundária pela regressão de Poisson com um estimador robusto. O risco relativo (RR) e o intervalo de confiança de 95% (IC 95%) foram calculados, e a significância estatística estabelecida pelo teste da razão de verossimilhança. O ajuste para as comparações múltiplas foi realizado pelo método sequencial de Bonferroni. Fatores de confusão foram selecionados entre características basais associadas com o método de tratamento no basal e com o tratamento farmacológico ótimo no seguimento (p<0,2). A magnitude da associação foi determinada pelo cálculo do RR, controlada por idade, sexo, cor de pele, anos de escolaridade, e diabetes mellitus no basal. Riscos relativos foram transformados em proporção de adesão por característica basal, e apresentado com seus IC95% correspondentes.

## Resultados

**Figura 1:**
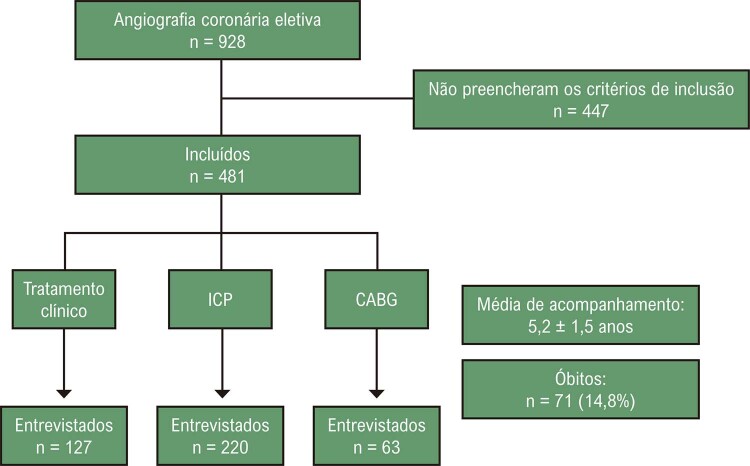
– Fluxograma do estudo. CABG: bypass da artéria coronária; ICP: intervenção coronária percutânea.

Entre os 928 pacientes submetidos à angiografia coronária eletiva, 481 preencheram os critérios de elegibilidade no basal. Desses, 415 (86,7%) pacientes apresentaram um SXscore baixo (>0 e ≤22) e 66 (13,7%) apresentaram um SXscore >22. Após uma média de 5,2 ± 1,5 anos de seguimento, 410 pacientes foram reavaliados, e 71 foram a óbito, 54 entre aqueles com SXscore baixo (13,1%) e 15 (22,6%) com um SXscore alto ( Figura 1 ).

**Figura 2:**
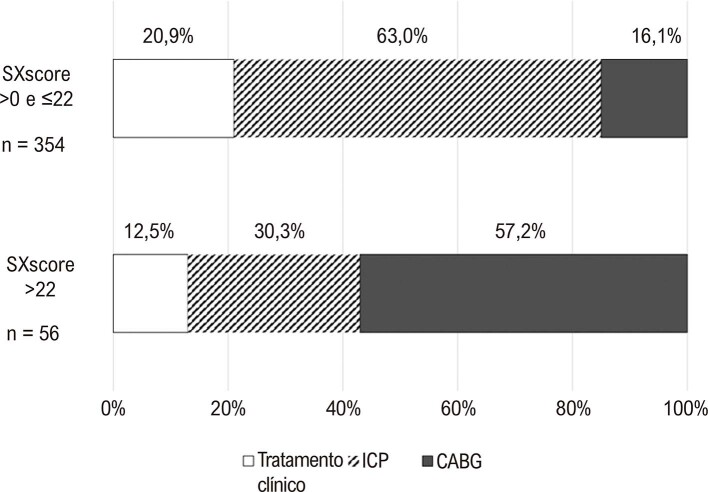
– Escolha do tratamento após cateterismo coronário diagnóstico e doença arterial coronariana confirmada de acordo com o SXscore calculado post-hoc. O valor de p para a interação foi <0.001. CABG: bypass da artéria coronária; ICP: intervenção coronária percutânea.

A Tabela 1 apresenta as características basais dos participantes, classificados de acordo com o método de tratamento. A maioria dos pacientes submetidos a CABG ou ICP era do sexo masculino, em comparação àqueles tratados exclusivamente com tratamento clínico (p<0,02). Os pacientes submetidos ao CABG apresentaram um SXscore significativamente mais alto em comparação àqueles submetidos à ICP ou à terapia clínica exclusiva (p<0,001). Não foram observadas outras diferenças estatisticamente significativas. A Figura 2 apresenta a proporção de métodos terapêuticos entre os participantes classificados pelo cálculo post-hoc do SXscore. Os pacientes com DAC moderada a grave foram submetidos principalmente à CABG, seguido por ICP, e somente aproximadamente 13% estavam sob terapia médica exclusiva. Por outro lado, os pacientes com DAC leve apresentaram maior probabilidade de serem tratados por ICP.

**Tabela 1:** – Características basais dos participantes do estudo, n (%) ou média ± DP

	Tratamento clínico (n=127)	ICP (n=220)	CABG (n=63)	Valor p
Idade (anos)	67,0 ± 9,8	66,4 ± 9,5	66,3 ± 7,5	0,8
Sexo masculino	69 (54,3)	153 (69,5)	41 (65,1)	0,02
Cor de pele branca	88 (69,3)	151 (68,6)	52 (82,5)	0,09
Anos de escolaridade ≥ 12	20 (15,7)	51 (23,2)	17 (27,0)	0,14
Índice de Massa Corporal (Kg/m ^2^ )	29,2 ± 4,9	28,0 ± 4,2	27,8 ± 4,2	0,37
Ex-fumante	85 (67,5)	145 (65,9)	34 (54,0)	0,16
Fumante atual	15 (11,8)	30 (13,6)	2 (3,2)	0,07
Hipertensão	118 (92,9)	208 (94,5)	60 (95,2)	0,8
Diabetes mellitus	41 (32,3)	58 (26,4)	26 (41,3)	0,07
Colesterol total (mg/dL)	175,7 ± 47,0	171,1 ± 44,5	175,2 ± 55,7	0,6
Colesterol total /HDL-c	4,4 ± 1,3	4,5 ± 1,2	4,5 ± 1,6	0,7
Insuficiência cardíaca	16 (13,3)	32 (14,5)	15 (23,8)	0,001
Fração de ejeção ventricular esquerda (%)*	62,1 ± 14,2	64,1 ± 13,3	59,2 ± 15,8	<0,001
Escore SYNTAX*	7,4 ± 9,1	9,3 ± 7,0	21,4 ± 9,5*	<0,001

CABG: bypass da artéria coronária; ICP: intervenção coronária percutânea; HDL-c: lipoproteína de alta densidade; DP: desvio padrão; *correção post-hoc de Bonferroni: p<0,001 entre CABG e ICP; CABG e tratamento clínico; e para ICP e tratamento clínico, p=NS.

**Figura 3:**
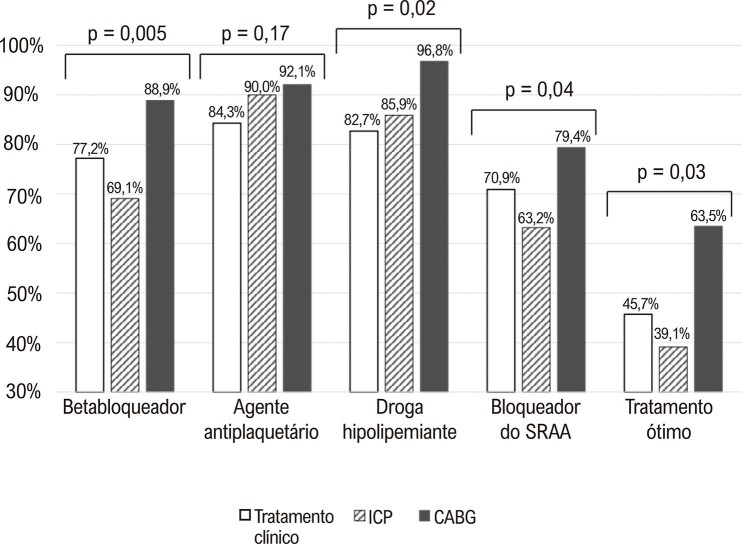
– Proporção de pacientes em terapia de prevenção secundária no seguimento de acordo com o tratamento inicial; CABG: bypass da artéria coronária; ICP: intervenção coronária percutânea; SRAA: sistema renina-angiotensina-aldosterona.

A proporção de pacientes que haviam recebido tratamento clínico ótimo foi maior entre os pacientes submetidos ao CABG que naqueles submetidos à ICP ou tratamento clínico exclusivo (p=0,003) ( Figura 3 ). Comparando-se individualmente, o uso de betabloqueadores, agentes hipolipemiantes, e bloqueadores do SRAA também foi significativamente mais frequente em pacientes submetidos ao CABG (p<0,05). Não foi observada diferença significativa quanto ao uso de agentes antiplaquetários.

**Figura 4:**
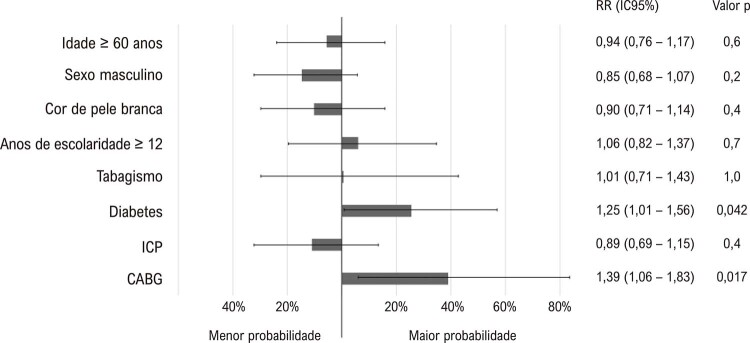
– Análise multivariada mostrando a probabilidade de pacientes em receberam tratamento farmacológico ótimo na avaliação de seguimento, controlada por fatores de confusão (idade, sexo, cor de pele, anos de escolaridade, tabagismo, diabetes, e procedimento inicial); CABG: bypass da artéria coronária; ICP: intervenção coronária percutânea.

Não se observou associação independente do tratamento clínico ótimo no seguimento com idade, sexo, cor de pele, anos de escolaridade ou tabagismo no momento basal. Por outro lado, pacientes que se submeteram ao CABG e pacientes diabéticos no basal apresentaram maior probabilidade de receberem tratamento farmacológico ótimo no seguimento (p = 0,017 e 0,042, respectivamente), independentemente da idade, sexo, cor de pele, escolaridade, e tabagismo no basal ( Figura 4 ).

## Discussão

Nesta coorte contemporânea de pacientes com DAC estável, encaminhados para diagnóstico por angiografia coronária, e que apresentaram DAC na angiografia anteriormente, a proporção de pacientes em tratamento clínico ótimo para prevenção secundária da DAC foi significativamente maior entre os tratados com CABG no basal, e comparação àqueles que receberam exclusivamente tratamento clínico ou ICP exclusiva. Após considerar os fatores de confusão, a associação persistiu, e os pacientes com diabetes no basal apresentaram maior probabilidade de receberem tratamento ótimo no seguimento, em comparação àqueles sem diabetes. Individualmente, betabloqueadores, drogas hipolipemiantes, e bloqueadores do SRAA foram usados com maior frequência por pacientes submetidos ao CABG. O uso de betabloqueadores e, especialmente, de bloqueadores do SRAA foi baixo em todos os pacientes.

A adesão a diretrizes de prevenção secundária é desejável a todos os pacientes com DAC, independentemente de revascularização, comorbidades, e outras características clínicas. ^3 - 5^ Estudos anteriores ^14 , 15 , 22^ mostraram taxas similares de adesão ao tratamento farmacológico ótimo para a prevenção secundária da DAC; no entanto, em todos eles, CABG foi associado à menor adesão, em contraste aos nossos resultados. No entanto, nossos resultados estão de acordo com uma metanálise de ensaios sobre revascularização, que mostrou uma diminuição na adesão global ao tratamento farmacológico ótimo sem uso de bloqueadores do SRAA de 67% em um ano para 53% em cinco anos. ^15^ Ao incluir os bloqueadores do SRAA, a adesão foi ainda mais baixa e diminuiu de 40% em um ano para 38% em cinco anos, sendo maior na ICP que no CABG em todos os tempos analisados. ^15^ Uma análise pós-hoc do ensaio SYNTAX mostrou que o tratamento farmacológico ótimo foi subutilizado em pacientes tratados com revascularização coronária, especialmente CABG. ^14^ Agentes antiplaquetários e drogas hipolipemiantes foram usados em mais de dois terços dos pacientes do estudo SYNTAX. Embora vários agentes possam ser utilizados na terapia antitrombótica preventiva o sistema de saúde público brasileiro fornece apenas clopidogrel e ácido acetilsalicílico, o que reduz o desafio da seleção do medicamento. ^23^ O uso dos bloqueadores do SRAA e de betabloqueadores foi consistentemente abaixo de 50%, ^14^ o que foi comparável aos estudos com pacientes tratados com CABG no ensaio PREVENT IV ^24^ e na alta hospitalar após uma síndrome coronária aguda descrito em um estudo polonês. ^22^ Um estudo brasileiro com pacientes com DAC estável também mostrou baixas taxas de tratamento farmacológico ótimo, especialmente de bloqueadores do SRAA, mas detectou diferenças significativas de acordo com sexo e sistema de saúde (se público ou privado). ^16^ Neste estudo, não se observou associação de sexo com tratamento farmacológico ótimo, e os pacientes incluídos eram todos do sistema de saúde público, em que agentes antiplaquetários, drogas hipolipemiantes, betabloqueadores e bloqueadores do SRAA são fornecidos gratuitamente. Assim, a acessibilidade não foi um impedimento para a prevenção secundária da DAC.

Diferenças na adesão ao tratamento farmacológico ótimo no seguimento podem ser explicadas pelo entendimento errôneo por parte dos pacientes e médicos de que doença grave (isto é, que requer cirurgia ou envolve diabetes) demanda maior cuidado intensivo, e vice-versa. Outro fator que deve ser levado em consideração é o medo de supermedicação. Ainda, o conceito de se usar agentes antiplaquetários e drogas hipolipemiantes está tradicionalmente relacionado à doença cardíaca no conhecimento popular, ao passo que o conceito do uso de betabloqueadores e bloqueadores do SRAA para prevenção secundária da DAC é mais recente e talvez menos difundido entre os médicos.

Vale mencionar que a DAC é uma doença sistêmica que envolve vários segmentos arteriais ^2^ e, portanto, o tratamento clínico ótimo é importante para reduzir sua progressão, bem como o risco de eventos cardiovasculares e mortalidade. ^14^ A adesão a todos os medicamentos para a prevenção secundária é desejável para todos os pacientes com DAC.

Nosso estudo teve limitações que devem ser consideradas. Primeiro, apesar de vários estudos haverem investigado a adesão à prevenção secundária, nosso estudo avaliou o acompanhamento dos pacientes que se submeteram angiografia coronária diagnóstica e suas terapias subsequentes, e estabeleceu o uso, em médio prazo, do tratamento ótimo para a prevenção secundária da DAC. No entanto, não conseguimos distinguir se os pacientes que haviam se submetido ao tratamento farmacológico ótimo eram não aderentes ao tratamento ou se eles não haviam recebido prescrição completa dos medicamentos. Contudo, este estudo descreve um cenário da vida real para a prevenção de eventos cardiovasculares futuros em pacientes vulneráveis. Segundo, estudos com acompanhamento de pacientes via entrevistas por telefone poderiam ser mais suscetíveis a viés que por visitas no consultório. Porém, as entrevistas foram conduzidas por um único cirurgião cardíaco treinado, que realizou a anamnese e processou as respostas dos pacientes ou familiares. Assim, provavelmente, o viés de medida não influenciou nos nossos resultados.

## Conclusão

A prevenção secundária da DAC é mais alta em pacientes submetidos ao CABG em comparação àqueles submetidos à ICP ou ao manejo clínico, e naqueles que apresentavam diabetes no momento do diagnóstico. Diferenças na adesão ao tratamento farmacológico ótimo nos estudos podem ser explicadas por uma concepção errônea por parte dos pacientes sobre tratamento invasivo da DAC e prevenção secundária subsequente. Estratégias para aumentar a adesão à prevenção secundária à DAC são necessárias.

## References

[1] (2018). Global Health Estimates 2016: Deaths by Cause, Age, Sex, by Country and by Region, 2000-2016.

[2] Polanczyk CA, Ribeiro JP (2009). Coronary Artery Disease in Brazil: Contemporary Management and Future Perspectives. Heart.

[3] Cesar LA, Ferreira JF, Armaganijan D, Gowdak LH, Mansur AP, Bodanese LC (2014). Guideline for Stable Coronary Artery Disease. Arq Bras Cardiol.

[4] Fihn SD, Blankenship JC, Alexander KP, Bittl JA, Byrne JG, Fletcher BJ (2014). 2014 ACC/AHA/AATS/PCNA/SCAI/STS Focused Update of the Guideline for the Diagnosis and Management of Patients with Stable Ischemic Heart Disease: A Report of the American College of Cardiology/American Heart Association Task Force on Practice Guidelines, and the American Association for Thoracic Surgery, Preventive Cardiovascular Nurses Association, Society for Cardiovascular Angiography and Interventions, and Society of Thoracic Surgeons. Circulation.

[5] Knuuti J, Wijns W, Saraste A, Capodanno D, Barbato E, Funck-Brentano C (2020). 2019 ESC Guidelines for the Diagnosis and Management of Chronic Coronary Syndromes. Eur Heart J.

[6] Iqbal J, Serruys PW (2017). Optimal Medical Therapy is Vital for Patients with Coronary Artery Disease and Acute Coronary Syndromes Regardless of Revascularization Strategy. Ann Transl Med.

[7] Boden WE, O’Rourke RA, Teo KK, Hartigan PM, Maron DJ, Kostuk WJ (2007). Optimal Medical Therapy with or Without PCI for Stable Coronary Disease. N Engl J Med.

[8] Stergiopoulos K, Brown DL (2012). Initial Coronary Stent Implantation with Medical Therapy vs Medical Therapy Alone for Stable Coronary Artery Disease: Meta-Analysis of Randomized Controlled Trials. Arch Intern Med.

[9] Maron DJ, Hochman JS, Reynolds HR, Bangalore S, O’Brien SM, Boden WE (2020). Initial Invasive or Conservative Strategy for Stable Coronary Disease. N Engl J Med.

[10] Okrainec K, Platt R, Pilote L, Eisenberg MJ (2005). Cardiac Medical Therapy in Patients After Undergoing Coronary Artery Bypass Graft Surgery: A Review of Randomized Controlled Trials. J Am Coll Cardiol.

[11] Hiratzka LF, Eagle KA, Liang L, Fonarow GC, LaBresh KA, Peterson ED (2007). Atherosclerosis Secondary Prevention Performance Measures After Coronary Bypass Graft Surgery Compared with Percutaneous Catheter Intervention and Nonintervention Patients in the Get With the Guidelines database. Circulation.

[12] Borden WB, Redberg RF, Mushlin AI, Dai D, Kaltenbach LA, Spertus JA (2011). Patterns and Intensity of Medical Therapy in Patients Undergoing Percutaneous Coronary Intervention. JAMA.

[13] Daly CA, De Stavola B, Sendon JL, Tavazzi L, Boersma E, Clemens F (2006). Predicting Prognosis in Stable Angina--Results from the Euro Heart Survey of Stable Angina: Prospective Observational Study. BMJ.

[14] Iqbal J, Zhang YJ, Holmes DR, Morice MC, Mack MJ, Kappetein AP (2015). Optimal Medical Therapy Improves Clinical Outcomes in Patients Undergoing Revascularization with Percutaneous Coronary Intervention or Coronary Artery Bypass Grafting: Insights from the Synergy Between Percutaneous Coronary Intervention with TAXUS and Cardiac Surgery (SYNTAX) Trial at the 5-Year Follow-Up. Circulation.

[15] Pinho-Gomes AC, Azevedo L, Ahn JM, Park SJ, Hamza TH, Farkouh ME (2018). Compliance with Guideline-Directed Medical Therapy in Contemporary Coronary Revascularization Trials. J Am Coll Cardiol.

[16] Birck MG, Goulart AC, Lotufo PA, Benseñor IM (2019). Secondary Prevention of Coronary Heart Disease: A Cross-Sectional Analysis on the Brazilian Longitudinal Study of Adult Health (ELSA-Brasil). Sao Paulo Med J.

[17] Fuchs FC, Ribeiro JP, Fuchs FD, Wainstein MV, Bergoli LC, Wainstein RV (2016). Syntax Score and Major Adverse Cardiac Events in Patients with Suspected Coronary Artery Disease: Results from a Cohort Study in a University-Affiliated Hospital in Southern Brazil. Arq Bras Cardiol.

[18] Almeida AS, Fuchs SC, Fuchs FC, Silva AG, Lucca MB, Scopel S (2020). Effectiveness of Clinical, Surgical and Percutaneous Treatment to Prevent Cardiovascular Events in Patients Referred for Elective Coronary Angiography: An Observational Study. Vasc Health Risk Manag.

[19] Campeau L (2002). The Canadian Cardiovascular Society Grading of Angina Pectoris Revisited 30 years later. Can J Cardiol.

[20] Sianos G, Morel MA, Kappetein AP, Morice MC, Colombo A, Dawkins K (2005). The SYNTAX Score: an angiographic tool grading the complexity of coronary artery Disease. EuroIntervention.

[21] Serruys PW, Morice MC, Kappetein AP, Colombo A, Holmes DR, Mack MJ (2009). Percutaneous Coronary Intervention versus Coronary-Artery Bypass Grafting for Severe Coronary Artery Disease. N Engl J Med.

[22] Jankowski P, Kosior DA, Sowa P, Szóstak-Janiak K, Kozieł P, Krzykwa A (2020). Secondary Prevention of Coronary Artery Disease in Poland. Results from the POLASPIRE Survey. Cardiol J.

[23] Bellettini E, De Luca L (2021). Antithrombotic Therapy in Patients with Coronary Artery Disease and Prior Stroke. J Clin Med.

[24] Harskamp RE, Alexander JH, Schulte PJ, Brophy CM, Mack MJ, Peterson ED (2014). Vein Graft Preservation Solutions, Patency, and Outcomes after Coronary Artery Bypass Graft Surgery: Follow-Up from the PREVENT IV Randomized Clinical Trial. JAMA Surg.

