# Supplementary material for: Prevenção Farmacológica Secundária da Doença Arterial Coronariana em Pacientes Submetidos ao Manejo Clínico, Intervenção Coronária Percutânea ou Cirurgia de Revascularização Miocárdica
Source: Arq Bras Cardiol. 2023 Feb 16;120(2):e20220403. [Article in Portuguese] doi: 10.36660/abc.20220403 (PMC12080629; doi:10.36660/abc.20220403)
Supplement: Translation

# Translation: Secondary Pharmacological Prevention of Coronary Artery Disease among Patients Submitted to Clinical Management, Percutaneous Coronary Intervention, or Coronary Artery Bypass Graft Surgery

**Authors:** Marcelo B. Lucca, Felipe C. Fuchs, Adriana S. Almeida, Marco V. Wainstein, Flavio D. Fuchs, Sandra C. Fuchs

**Affiliations:** 1 Brazil Programa de Pós-graduação em Cardiologia, Faculdade de Medicina, Universidade Federal do Rio Grande do Sul, Porto Alegre, RS – Brazil; 2 Brazil Instituto Nacional de Ciência e Tecnologia PREVER - Centro de Pesquisa Clínica - Hospital de Clínicas de Porto Alegre, Porto Alegre, RS – Brazil; 3 Brazil Divisão de Cardiologia - Hospital de Clínicas de Porto Alegre, Universidade Federal do Rio Grande do Sul, Porto Alegre, RS – Brazil

**Keywords:** Coronary Artery Disease, Coronary Artery Bypass, Percutaneous Coronary Intervention, Secondary Prevention, Drug Therapy

## Abstract

**Background:**

Secondary prevention is recommended for patients with evidence of coronary artery disease (CAD) regardless of the indication for treatment by coronary artery bypass graft surgery (CABG) or percutaneous coronary intervention (PCI).

**Objectives:**

This study evaluated whether clinical treatment, PCI or CABG had an influence on adherence to the pharmacological secondary prevention in patients with stable CAD.

**Methods:**

This cohort included patients aged ≥40 years with stable CAD confirmed by coronary angiography. The decision for medical treatment alone, or additionally with PCI or CABG, was made by the attending physicians. Adherence to the prescribed drugs recommended by the guidelines for secondary prevention (optimal pharmacological treatment), including antiplatelet agents, lipid-lowering drugs, beta-blockers, and renin-angiotensin-aldosterone system blockers, was assessed at follow-up. Differences were considered significant for p values <0.05.

**Results:**

From 928 patients enrolled at baseline, 415 had mild CAD and 66 moderate to severe CAD. The average follow-up was 5.2 ± 1.5 years. Patients submitted to CABG were more likely to receive the optimal pharmacological treatment than those submitted to PCI or treated clinically (63.5% versus 39.1% versus 45.7% respectively, p=0.003). Baseline factors independently associated with greater probability of having a prescription of optimal treatment at follow-up were CABG [39% higher (6% - 83%, p=0.017) and diabetes [25% higher (1% - 56%), p=0.042] than their counterparts treated by other methods and participants without diabetes, respectively.

**Conclusions:**

Patients with CAD submitted to CABG are more commonly treated with optimal pharmacological secondary prevention than patients treated by PCI or exclusively with medical therapy.

**Central Illustration:** : Secondary Pharmacological Prevention of Coronary Artery Disease among Patients Submitted to Clinical Management, Percutaneous Coronary Intervention, or Coronary Artery Bypass Graft Surgery

*CAD: coronary artery disease; CABG: coronary artery bypass graft; PCI: percutaneous coronary intervention.*

## Introduction

Cardiovascular disease has been the leading cause of death and burden of disease worldwide in the last 15 years. ^1^ Coronary artery disease (CAD), one of its presentations, affects 5% to 8% of Brazilians over 40 years of age. ^2^ Patients with clinical manifestations of CAD, such as angina pectoris, myocardial infarction, or evidence of lesions on coronary angiography, are candidates for secondary prevention. Guidelines recommend the use of antiplatelet agents, lipid-lowering drugs, beta-blockers, and renin-angiotensin-aldosterone system (RAAS) blockers, all of them with high levels of evidence. ^3 - 5^

Revascularization performed by coronary artery bypass grafting (CABG) improves survival in patients with unprotected left main stem disease, three-vessel CAD, or diabetes, particularly for those with severe symptoms, early positive noninvasive tests, or impaired left ventricular function. ^6^ Percutaneous coronary intervention (PCI) is usually preferred for individuals without a clear indication for CABG, whose symptoms persist despite of pharmacological treatment. ^3^ Large randomized trials, comparing the initial invasive or conservative strategies for patients with stable CAD, did not find significant differences in cardiovascular events and mortality. ^6 - 9^

Regardless of revascularization, pharmacological management remains the standard treatment for secondary prevention of CAD. ^3 - 5^ However, adherence has been suboptimal in many settings. ^10 - 12^ In the Euro Heart Survey, a considerable proportion of individuals with stable CAD managed medically or invasively were not on optimal pharmacological treatment, and this was associated with worse outcomes. ^13^ A post-hoc analysis of the Synergy Between PCI with Taxus and Cardiac Surgery (SYNTAX) trial additionally showed that the proportion of patients for whom optimal pharmacological treatment had been prescribed was 41% at discharge of revascularization, and dropped to one-third after five years. ^14^ In a metanalysis of studies on coronary revascularization, optimal pharmacological treatment decreased from 40% at one-year follow-up to 38% at five years, and percentages remained higher in PCI than in CABG at all time points. ^15^ Data also suggest a correlation between the differences in adherence and clinical outcomes when comparing PCI and CABG at five years. A study conducted in Brazil detected differences in optimal pharmacological treatment between low versus high-income individuals. ^16^ To our knowledge, the association between the type of treatment, CABG, and PCI, or exclusively medical adherence to secondary prevention was not assessed in a contemporary cohort. The purpose of this report was to assess if the method of treatment of stable CAD – CABG, PCI, or exclusive medical treatment – influenced the adherence to the optimal pharmacological treatment for secondary prevention of CAD.

## Methods

Participants of this report were evaluated in a cohort study designed to assess several outcomes in patients with stable CAD. ^17 , 18^ Distribution of deaths and other major adverse cardiovascular events (MACE) at follow-up, according to initial treatment, was previously reported elsewhere. ^17 , 18^

The cohort included men and women, aged ≥40 years, with stable and angiographically significant CAD. Patients were referred for elective coronary angiography due to clinical suspicion of CAD ^19^ with or without evidence of ischemia in noninvasive tests. At baseline, individuals with acute coronary syndrome, previous revascularization (CABG or PCI), chronic renal disease, previous or current cancer diagnosis, severe psychiatric disease, or no evidence of significant CAD (SYNTAX score [SXscore]<1) were excluded.

At baseline, traditional cardiovascular risk factors, socioeconomic and demographic factors, lifestyle, and previous morbidity data were assessed during a face-to-face interview using a standardized questionnaire. Trained research assistants performed blood pressure (BP) and anthropometric measurements at enrolment, prior to the index catheterization. Hypertension was defined systolic BP ≥140 mmHg, diastolic BP ≥90 mmHg, or use of BP-lowering drugs. Body mass index (BMI) (weight [kg] /height [m ^2^ ]) was categorized as <25, 25–29, or ≥30 kg/m ^2^ .

Laboratory assessments were performed after 12 hours of fasting. Blood samples were withdrawn from the femoral artery sheath immediately after cardiac catheter insertion but before heparin administration. Diabetes mellitus was characterized by fasting glucose ≥126 mg/dL or use of antidiabetic agents. Hypercholesterolemia was characterized by a total cholesterol level ≥200 mg/dL or use of lipid-lowering drugs. Coronary angiography at the index catheterization was performed by experienced interventional cardiologists through radial or transfemoral accesses. Significant CAD was diagnosed by quantitative analysis of the major epicardial vessels (e.g., the left main coronary artery, anterior descending artery, circumflex artery, right coronary artery, and vessels with diameters ≥2.5 mm), diagonal branches, obtuse marginal artery, posterolateral branches, and posterior descending artery. ^17^ Significant CAD was defined by the presence of at least one major epicardial coronary artery presenting a stenosis ≥50%. The SXscore was calculated for each affected artery, and the scores were added to provide the patient’s final SXscore. ^20^ An SXscore ≤22 was categorized as mild CAD and scores higher than 22 were categorized as moderate to severe CAD. ^21^ Two interventional cardiologists independently evaluated a subsample of images, and quality control was made by a third physician who assessed interobserver variation. The attending physicians, who did not participate in the study, received the images and a coronary angiography report, but were unaware of additional SXscores. The decision between CABG, PCI, or exclusive medical treatment was defined by the attending physicians, based on previous training in cardiology, but non-standardized usual clinical practice. The attending physicians used to discuss complex cases with the interventional cardiologists and surgeons who had performed the diagnostic procedure.

At follow-up, participants were invited by phone to be interviewed by a trained physician. A standardized questionnaire was used to record the treatment performed after index coronary angiography, subsequent comorbidities, hospital admissions, current medical treatment, and general health status. Adherence to optimal pharmacological treatment was defined as the reported use of all medications recommended for secondary prevention for which patients were eligible, including antiplatelet agents, statins or other lipid-lowering drugs, beta-blockers, and RAAS blockers, which are provided by the Brazilian public healthcare system.

### Sample size and statistical analysis

The sample size was calculated to test the primary hypothesis, ^17^ which had 80% power and 0.05 significance level (two-tailed) to detect a hazard ratio of at least 2.4, considering that 5% of patients with a low SXscore and 12% with a high SXscore would present MACE. In this additional analysis, we included only surviving participants who had a baseline SXscore >0 and were, therefore, eligible for secondary prevention. All analyses were conducted using the Statistical Package for the Social Sciences (SPSS; version 22.0; IBM corp., Armonk, NY, USA). Differences were considered significant for a P value <0.05. Data normality was verified using the boxplot and the Shapiro-Wilk test. Continuous variables were presented as mean ± standard deviation (SD), and categorical variables were presented as absolute numbers with percentages and confidence intervals when relevant. Baseline characteristics were analyzed using the analysis of variance (one-way ANOVA with and Bonferroni post-hoc test) for continuous variables and the chi-square test for categorical variables to compare clinical treatment, PCI and CABG. Chi-square tested the proportion of patients treated with optimal pharmacological treatment between participants treated with exclusive medical therapy with those who were additionally treated with CABG or PCI. In addition, we explored the association of several baseline characteristics with adherence to secondary prevention by Poisson regression with a robust estimator. Relative risk (RR) and 95% confidence interval (CI) were calculated and statistical significance was established by the likelihood ratio test. Adjustment for multiple comparisons was done by the Sequential Bonferroni test. Confounding factors were selected among baseline characteristics associated with the method of treatment at baseline and optimal pharmacological treatment at follow-up (p value <0.2). The magnitude of the association was determined by calculating the RR, controlling for age, sex, skin color, years of schooling, current smoking, and diabetes mellitus at baseline. Relative risks were transformed into proportion of adherence by baseline characteristic and presented with their corresponding 95% CIs.

## Results

Among 928 patients undergoing elective coronary angiography, 481 fulfilled the eligibility criteria at baseline. Of these, 415 (86.7%) patients had a low SXscore (>0 and ≤22) and 66 (13.7%) had SXscore >22. After 5.2 ± 1.5 years mean follow-up, 410 patients were further evaluated, and 71 patients died, 54 among patients with low SXscore (13.1%) and 15 (22.6%) with high SXscore ( Figure 1 ).

**Figure 1:** – Study flow diagram. CABG: coronary artery bypass graft; PCI: percutaneous coronary intervention.

Table 1 presents the baseline characteristics of the participants according to the method of treatment. Patients submitted to CABG or PCI were mostly male, compared to those treated exclusively with medical therapy (p<0.02). Patients treated by CABG had significantly higher SXscore compared to PCI or exclusive medical therapy (p<0.001). No other statistically significant differences were observed. Figure 2 shows the proportion of therapeutic methods among participants classified by the post hoc calculation of SXscore. Patients with moderate to severe CAD were mostly submitted to CABG, followed by PCI, and only approximately 13% were under exclusive medical therapy. On the other hand, patients with mild CAD were more likely to have been treated by PCI.

**Table 1:** – Characteristics of study participants at baseline, n (%) or mean ± SD

	Clinical (n=127)	PCI (n=220)	CABG (n=63)	p value
Age (years)	67.0 ± 9.8	66.4 ± 9.5	66.3 ± 7.5	0.8
Male sex	69 (54.3)	153 (69.5)	41 (65.1)	0.02
White skin color	88 (69.3)	151 (68.6)	52 (82.5)	0.09
Years of schooling ≥ 12	20 (15.7)	51 (23.2)	17 (27.0)	0.14
Body mass index (kg/m ^2^ )	29.2 ± 4.9	28.0 ± 4.2	27.8 ± 4.2	0.37
Ever smoked	85 (67.5)	145 (65.9)	34 (54.0)	0.16
Current smoking	15 (11.8)	30 (13.6)	2 (3.2)	0.07
Hypertension	118 (92.9)	208 (94.5)	60 (95.2)	0.8
Diabetes mellitus	41 (32.3)	58 (26.4)	26 (41.3)	0.07
Total cholesterol (mg/dL)	175.7 ± 47.0	171.1 ± 44.5	175.2 ± 55.7	0.6
Total cholesterol/HDL-c ratio	4.4 ± 1.3	4.5 ± 1.2	4.5 ± 1.6	0.7
Heart failure	16 (13.3)	32 (14.5)	15 (23.8)	0.001
Left ventricular fraction ejection (%)*	62.1 ± 14.2	64.1 ± 13.3	59.2 ± 15.8	<0.001
SYNTAX score*	7.4 ± 9.1	9.3 ± 7.0	21.4 ± 9.5*	<0.001

PCI: percutaneous coronary intervention; CABG: coronary artery bypass graft; HDL-c: high-density lipoprotein cholesterol; SD: standard deviation. * Post-hoc analysis using Bonferroni test: P<0.001 between CABG and PCI; CABG and clinical treatment; and for PCI and clinical treatment (P=NS).

**Figure 2:** – Choice of treatment after diagnostic coronary catheterization and confirmed coronary artery disease, according to the post-hoc calculated SXscore. P-value for interaction <0.001. CABG: coronary artery bypass graft; PCI: percutaneous coronary intervention.

The proportion of patients who were being treated with the optimal medical treatment was higher among those submitted to CABG than in those submitted to PCI or exclusive medical treatment (p=0.003) ( Figure 3 ). When compared individually, the use of beta-blockers, lipid-lowering drugs, and RAAS blockers were also significantly more frequent in patients submitted to CABG (p<0.05). No significant difference was found regarding the use of antiplatelet agents.

**Figure 3:** – Proportion of patients under secondary prevention therapy at follow-up according to the index treatment; CABG: coronary artery bypass graft; PCI: percutaneous coronary intervention; RAAS: renin-angiotensin-aldosterone system.

There was no independent association of optimal medical treatment at follow-up with age, sex, skin color, years of schooling or current smoking at baseline. In contrast, patients who underwent CABG and those who were diabetic at baseline had a higher probability of being under optimal pharmacological treatment at follow-up (p = 0.017 and 0.042, respectively), independently of age, sex, skin color, years at schooling, and current smoking at baseline ( Figure 4 ).

**Figure 4:** – Multivariable analysis showing the probability of patients being under optimal pharmacological treatment at the follow-up assessment, controlling for confounding factors (age, sex, skin color, years at school, current smoking, diabetes, and index procedure); CABG: coronary artery bypass graft; PCI: percutaneous coronary intervention.

## Discussion

In this contemporary cohort of patients with stable CAD, referred for diagnostic coronary angiography, and who had angiographically significant CAD, the proportion of patients under optimal medical treatment for secondary prevention of CAD was significantly higher among those treated with CABG at baseline, compared to those who received exclusive medical therapy or PCI. After considering confounding factors, the association persisted, and patients with diabetes at baseline had a higher probability of being treated with optimal treatment at follow-up than patients without diabetes. Individually, beta-blockers, lipid-lowering drugs, and RAAS blockers were more frequently used among CABG patients. The use of beta-blockers and especially of RAAS blockers was low in all patients.

Adherence to secondary prevention guidelines is desirable for all patients with CAD, regardless of revascularization, comorbidities, and other clinical characteristics. ^3 - 5^ Previous studies ^14 , 15 , 22^ have shown similar overall rates of adherence to optimal pharmacological treatment for secondary prevention of CAD, however, in all of them CABG was associated with lower adherence, in contrast to the results of this report. However, our results are in accordance with a meta-analysis of contemporary revascularization trials which showed that overall adherence to optimal pharmacological treatment without RAAS blockers decreased over time from 67% at one year to 53% at five years. ^15^ When including RAAS blockers, adherence was even lower and decreased from 40% at one year to 38% at five years, and was higher in PCI than in CABG at all time points. ^15^ A post hoc analysis of the SYNTAX trial showed that optimal pharmacological treatment was underutilized in patients treated with coronary revascularization, especially CABG. ^14^ Antiplatelet agents and lipid-lowering drugs were used in more than two-thirds of the SYNTAX patients. Despite several drugs can be used in preventive antithrombotic therapy, the Brazilian public health care system provides clopidogrel and acetylsalicylic acid only, which avoids the challenge of drug selection. ^23^ The use of RAAS blockers and beta-blockers was consistently below 50%, ^14^ which was comparable to the reports of patients treated with CABG in the PREVENT IV trial ^24^ and at discharge after acute coronary syndrome in study conducted in Poland. ^22^ A Brazilian study with patients with stable CAD also showed low rates of optimal pharmacological treatment, especially of RAAS blockers, but detected significant differences according to sex and health care system (public vs. private). ^16^ In this report, sex was not associated with optimal pharmacological treatment, and patients included were all from the public health care system, where antiplatelet agents, lipid-lowering drugs, beta-blockers and RAAS blockers are provided free of charge. Therefore, affordability was not a deterrent to secondary prevention of CAD.

Differences in adherence to optimal pharmacological treatment at follow-up may be explained by misconceptions on the part of patients and physicians that severe disease (i.e., that requires surgery or involves diabetes) would require more intensive care, and vice versa. Another factor that should be taken into consideration is the fear of overmedicating. Furthermore, the concept of using antiplatelet and lipid-lowering drugs is traditionally linked to heart disease in the popular knowledge, while the concept of using beta-blockers and RAAS blockers for secondary prevention of CAD is more recent and maybe less spread among physicians.

It is worth mentioning that CAD is a systemic disease that involves multiple arterial segments ^2^ and thus, optimal medical treatment is important in reducing its progression, the risk of cardiovascular events and mortality. ^14^ Adherence to the full set of drugs for secondary prevention is desirable for all patients with CAD.

Our study had limitations that need to be addressed. First, although several studies have investigated adherence to secondary prevention, our study assessed the follow-up of patients who had undergone elective diagnostic coronary angiography and their subsequent therapies and established the mid-term use of optimal treatment for secondary prevention of CAD. However, we were unable to determine whether those who had not undergone optimal pharmacological treatment were nonadherent or whether they had not received full prescriptions of these drugs. Nevertheless, this report describes a real-life scenario for preventing future cardiovascular events among vulnerable patients. Second, follow-up investigations through phone interviews could be more susceptible to bias than office visits. Nonetheless, interviews were conducted by a single trained cardiac surgeon who was able to perform an anamnesis and correctly process the answers of patients or relatives. Therefore, measurement bias was unlikely to play a role in our results.

## Conclusion

Secondary prevention of CAD is higher in patients submitted to CABG compared to clinical management or PCI, and in those who had diabetes at the time of diagnosis. Differences in reported adherence to optimal pharmacological treatment may be explained by misconceptions on the part of patients regarding invasive treatment of CAD and subsequent secondary prevention. Strategies to increase adherence to secondary prevention for CAD are warranted.
